# Microwave Ablation Combined with Flt3L Provokes Tumor‐Specific Memory CD8^+^ T Cells‐Mediated Antitumor Immunity in Response to PD‐1 Blockade

**DOI:** 10.1002/advs.202413181

**Published:** 2024-12-04

**Authors:** Meixiang Wang, Jing Sang, Fengkuo Xu, Shulong Wang, Peng Liu, Ji Ma, Zhengtao Chen, Qi Xie, Zhigang Wei, Xin Ye

**Affiliations:** ^1^ Department of Oncology The First Affiliated Hospital of Shandong First Medical University & Shandong Provincial Qianfoshan Hospital Shandong Provincial Lab for Clinical Immunology Translational Medicine in Universities Shandong Lung Cancer Institute 16766 Jingshi Road Jinan 250014 P. R. China; ^2^ Department of Pathology Shandong Provincial Third Hospital 11 Wuyingshan Zhonglu Road Jinan 250100 P. R. China; ^3^ Shandong Academy of Preventive Medicine Shandong Center for Disease Control and Prevention 16992 Jingshi Road Jinan 250014 P. R. China; ^4^ School of Laboratory Animal & Shandong Laboratory Animal Center Shandong First Medical University & Shandong Academy of Medical Sciences 6699 Qingdao Road Jinan 250014 P. R. China; ^5^ Cheeloo College of Medicine Shandong University 27 Shanda Nanlu Road Jinan 250100 P. R. China

**Keywords:** dendritic cells, ICOS‐ICOSL axis, microwave ablation, non‐small cell lung cancer, tumor‐specific memory CD8^+^ T cells

## Abstract

For medically inoperable non‐small cell lung cancer, microwave ablation (MWA) represents a super minimally invasive alternative treatment. However, tumor recurrence remains a concern. Here, it is demonstrated that the combination of MWA with Flt3L significantly inhibits tumor recurrence by CD8^+^ central memory T (T_CM_)‐like cell‐dependent antitumor immune responses within the tumor‐draining lymph nodes (TdLN). TdLN‐T_CM_‐like cells encompassed both tumor‐specific memory T (T_TSM_) and progenitor‐exhausted T (T_PEX_) cells. The expansion of these cells markedly altered the differentiation of exhausted T cells within the tumor microenvironment (TME). T_PEX_ predominantly differentiated into transitory effector‐like exhausted T cells (T_EX_‐int). The expansion of T_TSM_ cells elicited by the combined therapy was reliant on conventional dendritic cells (cDCs) and was likely specifically dependent on the migratory cDC1s (Mig cDC1s) within the TdLN. The upregulation of ICOSL on migratory cDC1s was pivotal in initiating T_TSM_‐like cell‐mediated antitumor responses. *Slc38a2* may be a critical gene responsible for the upregulation of ICOSL in Mig cDC1s following combined treatment. Finally, the combined treatment significantly enhanced the antitumor efficacy of immunotherapy based on PD‐1 blockade. The research thereby afforded a novel strategic approach to forestall tumor recurrence after MWA therapy, while also providing the foundational proof‐of‐concept for impending clinical investigations.

## Graphical abstract

MWA combined with Flt3L can reshape TME from “cold” to “hot” in LLC murine model. Compared with MWA monotherapy, combinational therapy significantly induces the differentiation of T_CM_‐like and T_TSM_/T_PEX_ cells within TdLN, mediating prominent antitumor immunity in response to PD‐1 blockade. Further exploration reveals *Slc38a2* expression is increased in Mig cDC1s, leading to enhanced DC functionality characterized by ICOSL upregulation.

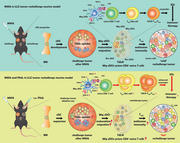

## 1 Introduction

Globally, non‐small cell lung cancer (NSCLC) is the predominant cause of cancer‐related death,^[^ ^1^ ^]^ and surgical resection remains the standard therapy.^[^ ^2^ ^]^ Indeed, 25–30% of patients are at high risk for surgery, such as those with poor cardiopulmonary function and elderly individuals.^[^ ^3^, ^4^ ^]^ Percutaneous image‐guided thermal ablation (IGTA) is a super minimally invasive treatment option for medically inoperable patients that spares lung parenchyma and has been endorsed by multiple professional societies.^[^ ^2^, ^5^, ^6^, ^7^, ^8^, ^9^ ^]^ IGTA techniques include radiofrequency ablation (RFA), microwave ablation (MWA), cryoablation, and laser ablation. MWA has several potential advantages over RFA, including faster ablation, higher temperature, less sensitivity to tissue types with more consistent results, relative insensitivity to “heat sinks,” and the ability to create a much larger ablative zone, which is more suitable for ablation of lung tumors.^[^ ^10^, ^11^ ^]^ MWA is a safe and effective treatment option for both early‐stage and advanced lung cancer, playing a significant role in the management of NSCLC.^[^ ^12^, ^13^, ^14^ ^]^ Despite its notable advantages, tumor recurrence following MWA remains a significant and stubborn challenge. The local recurrence rates, which range from 9 to 37%, severely threaten the prognosis and lifespan of cancer patients.^[^ ^15^, ^16^ ^]^ Thus, there is an immediate demand for the identification of an efficacious therapeutic strategy to enhance the treatment outcome.

Immunotherapy, by harnessing the patient's immune system to target tumor cells, demonstrates considerable promise in preventing tumor regression and the spread of cancer to distant sites, and it is currently transforming the landscape of treatment for various tumor types.^[^ ^17^, ^18^, ^19^ ^]^ MWA can provoke a rapid dispersal of tumor debris, which includes various “danger signals” such as tumor‐associated antigens (TAAs),^[^ ^20^, ^21^ ^]^ and it can also attract a variety of immune cells, including dendritic cells (DCs), macrophages, natural killer (NK) cells, and T cells, to the treated tumor.^[^ ^22^ ^]^ Although the antitumor immune response evoked by MWA is insufficient to prevent tumor relapse and systemic dissemination,^[^ ^23^ ^]^ it nonetheless establishes a congenial environment for the engagement of tumor‐specific antigens with immune cells, thereby laying the foundation for immunotherapeutic interventions.

Recently, an array of local and systemic immunotherapeutic approaches has been integrated post‐MWA to synergistically augment oncotherapy, including Immune checkpoint inhibitors (ICIs).^[^ ^24^ ^]^ ICIs have shown promise in enhancing antitumor immune responses when combined with MWA for NSCLC treatment, providing benefits to patients.^[^ ^25^ ^]^ Nevertheless, given that ICIs primarily function by reinstating T‐cell activity within the tumor microenvironment (TME),^[^ ^26^ ^]^ the less infiltration of immune cells in “cold” tumors hinders the effectiveness of combining MWA with ICIs for the management of NSCLC, still posing a risk of recurrence.^[^ ^25^ ^]^ In such scenarios, turning nonresponsive “cold” tumors into responsive “hot” ones serves as a pivotal strategy for preventing recurrence after MWA therapy.

Immunologically “cold” tumors are distinguished by their absence of inflammation and T cells, whereas “hot” tumors are characterized by signatures of immune activation and marked T‐cell infiltration.^[^ ^27^ ^]^ Emerging research indicates that the density of dendritic cells (DCs) is positively correlated with the recruitment of T cells, prolonged overall survival, and a favorable clinical response to ICIs.^[^ ^28^, ^29^ ^]^ DCs act as a critical bridge between innate and adaptive immune responses, encompassing various subsets, such as plasmacytoid DCs (pDCs) and conventional DCs (cDCs), with cDCs further subdivided into CD8α^+^ or CD103^+^ cDC1s and CD11b^+^ cDC2s.^[^ ^30^ ^]^ Dendritic cell–T‐cell crosstalk is essential for generating effective antitumor T‐cell immunity.^[^ ^31^ ^]^ Certainly, cDC1s can phagocytose and process TAAs in the TME and then migrate to tumor‐draining lymph nodes (TdLN), where they cross‐present TAAs to CD8^+^ T cells through the major histocompatibility complex class I (MHC‐I), initiating antitumor immune responses.^[^ ^32^, ^33^, ^34^ ^]^ Moreover, cDC1s are not only crucial for activating CD8^+^ T cells but also indispensable for maintaining their niches.^[^ ^35^, ^36^ ^]^ These suggest that DCs could be pivotal in converting “cold” tumors into “hot” ones, which is a critical step in their responsiveness to immunotherapy.

The growth factor Flt3 ligand (Flt3L) plays a pivotal role in DC homeostasis and development by controlling the survival and expansion of DCs through binding to the Flt3 receptor tyrosine kinase on their surface.^[^ ^37^ ^]^ Previous studies have demonstrated that intravenous administration of Flt3L can promote the expansion of DCs and enhance the infiltration of tumor‐specific CD8^+^ T cells into tumors.^[^ ^38^, ^39^, ^40^, ^41^, ^42^, ^43^ ^]^ A recent study showed that combining irreversible electroporation ablation with Flt3L and CD40Leffectively increased T‐cell infiltration in the TME, activated antitumor immune responses, and reduce the recurrence of pancreatic cancer.^[^ ^44^ ^]^ However, it remains unclear whether the combination of MWA and Flt3L treatment can reshape the TME of NSCLC by increasing the number of DCs and promoting the cross‐presentation of TAAs to CD8^+^ T cells, thereby enhancing antitumor immune responses and reducing recurrence. Furthermore, the main focus of Flt3L research has been its potential to generate DCs from bone marrow progenitors, with a limited understanding of how these cells influence CD8^+^ T‐cell niches.^[^ ^45^ ^]^ Unraveling the molecular mechanisms by which DCs preserve niches for CD8^+^ T cells will offer novel immunotherapy targets for reshaping the TME of NSCLC through the integration of MWA.

In this study, we explored the impact of combined MWA and Flt3L treatment on NSCLC via a syngeneic murine tumor rechallenge model, the subcutaneous injection of Lewis lung carcinoma (LLC) cells. The synergistic approach of MWA and Flt3L triggered a potent tumor‐specific memory T (T_TSM_) cell response. This led to the transformation of “cold” tumors into “hot” ones, resulting in improved survival post‐MWA and enhanced protection against rechallenged tumors in mice. A key mechanism underlying this strategy is the enhancement of ICOS‐related signals, which facilitate communication between cDC1s and T_TSM_ cells in the TdLN. The combined treatment significantly enhances the antitumor efficacy of immunotherapy based on PD‐1 blockade. This research has profound implications for advancing the field of cancer immunotherapy and enhancing outcomes in patients with this challenging disease.

## 2 Results

### 2.1 MWA Plus Flt3L Markedly Attenuates Tumor Recurrence Post‐Ablation

**Figure 1:**
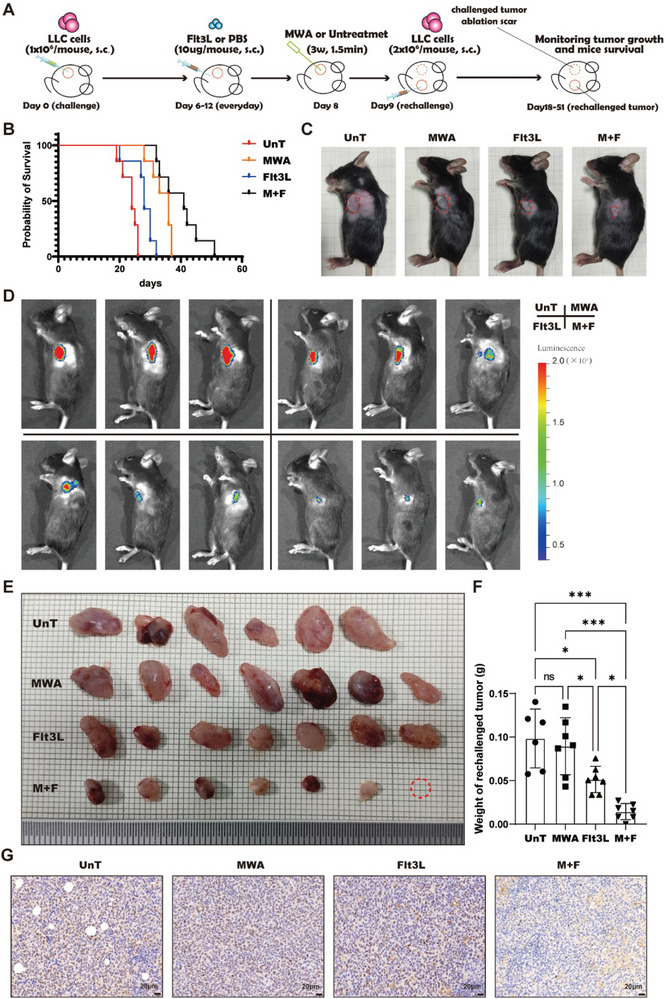
The combination of MWA and Flt3L treatment elicits a synergistic inhibition of the growth in rechallenged tumors. A) Schematic illustration of the experimental design for B‐G. B) Survival of mice in the LLC tumor rechallenge model. C) Observation of tumors in the LLC tumor rechallenge model. D) Visualization of tumor growth via bioluminescence imaging in the LLC tumor rechallenge model. E) Images of the rechallenged tumors in all treatment groups. F) The statistical analysis delineating the quantification of rechallenged tumor weights across diverse control cohorts. G) Ki67 immunohistochemical staining in all treatment groups. Scale bars, 20 µm. n = 6–8 mice per group; data were acquired from at least two independent experiments. Statistical significance was assessed via one‐way ANOVA followed by Tukey's multiple comparisons test, and survival analysis was conducted via the log‐rank test in GraphPad Prism. 9.0.0 software (^*^ *p* < 0.05, ^**^ *p* < 0.01, ^***^ *p* < 0.001). “UnT” refers to untreated control, “MWA” refers to MWA‐treated control, “Flt3L” refers to Flt3L‐treated control, and “M+F” refers to the group that received treatment with MWA combined with Flt3L.

To assess the antitumor effects of a combined treatment approach involving MWA and Flt3L, we established a LLC tumor rechallenge murine model (**Figure** **1**A). Compared with those in the other control groups, the survival of the mice in the cohort receiving the combination of MWA and Flt3L treatment was prolonged (Figure 1B). Moreover, the growth of the rechallenged tumors in the combined treatment group was significantly slower than that in other groups (Figure 1C). Tumor progression was tracked by capturing bioluminescence signals emitted by LLC‐Luc cells, revealing notably decreased growth of the rechallenged tumors in the combined treatment group compared with the others (Figure 1D). After the mice were euthanized and the tumors were dissected, substantially lower weights of the rechallenged tumors in the combined treatment group than other groups were observed (Figure 1E,F). The Ki67 expression level in the rechallenged tumors was lower in the combined treatment group than other groups (Figure 1G). These findings indicate that combined modality therapy incorporating MWA with Flt3L has robust synergistic antitumor efficacy, displaying improved survival outcomes and significant inhibition on tumor recurrence following ablation in a murine model.

### 2.2 CD8^+^ T Cells Play a Pivotal Role in the Inhibition of Tumor Recurrence

**Figure 2:**
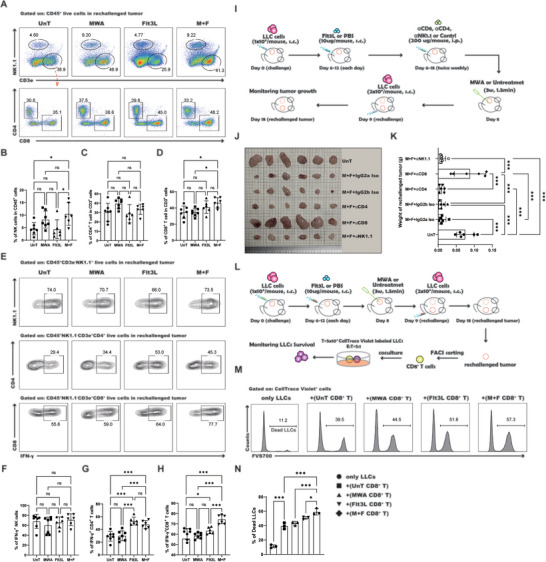
CD8^+^ T cells are pivotal in mediating antitumor immunity following the combined treatment. A) Flow cytometric analysis of the ratios of NK cells, CD4^+^, and CD8^+^ T cells in the tumor on day 18 following various treatment regimens. B) Statistical findings for the ratios of NK cells. C) Statistical findings for the ratios of CD4^+^ T cells. D) Statistical findings for the ratios of CD8^+^ T cells. E) Flow cytometric analysis of IFN‐γ secretion by NK cells, CD4^+^ and CD8^+^ T cells. F) Statistical findings for the ratios of IFN‐γ^+^ NK cells. G) Statistical findings for the ratios of IFN‐γ^+^ CD4^+^ T cells. H) Statistical findings for the ratios of IFN‐γ^+^ CD8^+^ T cells. I) Schematic illustration of the experimental design for J‐K. J) Images of the rechallenged tumors in all groups. K) The statistical analysis delineates the quantification of rechallenged tumor weights across diverse control cohorts. L) Schematic illustration of the experimental design for the coculture assay used to evaluate the cytotoxicity of antitumor CD8^+^ T cells via flow cytometry. M) Representative flow cytometry plots showing the percentage of dead LLCs in cocultures. N) Statistical results for M. Each group consisted of 6–8 mice, and data were acquired from at least two independent experiments. Statistical significance was determined via one‐way ANOVA followed by Tukey's multiple comparisons tests, all of which were conducted with GraphPad Prism 9.0.0 software (^*^ *p* < 0.05, ^**^ *p* < 0.01, ^***^ *p* < 0.001). “UnT” signifies untreated group. “M+F+IgG2a Iso” signifies MWA, Flt3L, and IgG2a isotype control‐treated group. “M+F+IgG2b Iso” signifies MWA, Flt3L, and IgG2b isotype control‐treated group. “M+F+αCD4” signifies MWA, Flt3L, and anti‐CD4 antibody‐treated group. “M+F+αCD8” signifies MWA, Flt3L, and anti‐CD8 antibody‐treated group. "M+F+αNK1.1" signifies MWA, Flt3L and anti‐NK1.1 antibody‐treated group.

NK cells, CD8^+^ cytotoxic T cells (CTLs), and CD4^+^ T cells have been recognized as the principal immune surveillance cells orchestrating the antitumor immune response.^[^ ^46^, ^47^, ^48^, ^49^, ^50^ ^]^ To delineate the key cellular players in antitumor immunity, we employed flow cytometry to meticulously evaluate the alterations in NK cells, CD4^+^, and CD8^+^ T cells within the TME following treatment with MWA synergized with Flt3L administration. Our findings demonstrated a substantial elevation in the relative abundance of both NK cells and CD8^+^ T cells (**Figure** **2**A,B,D), whereas the prevalence of CD4^+^ T cells remained largely unchanged (Figure 2A,C). Moreover, we conducted a detailed analysis of IFN‐γ secretion by NK cells, CD4^+^, and CD8^+^ T cells. Our findings demonstrated a notable upsurge in IFN‐γ production by both CD4^+^ and CD8^+^ T cells after the integrative therapeutic intervention (Figure 2E,G,H). In contrast, the IFN‐γ secretion by NK cells did not exhibit a significant change (Figure 2E,F). These findings implicate that the synergistic therapeutic regimen alters the ratio or functional status of NK, CD4^+^, and CD8^+^ T cells, each of which may play a pivotal role in mediating the antitumor immune response facilitated by the integrated treatment strategy. Thus, to delineate the pivotal cellular mediators underlying the antitumor efficacy of the combined therapeutic regimen, we utilized the LLC tumor rechallenge murine model to systematically deplete NK cells, CD4^+^ and CD8^+^ T cells, enabling the assessment of their distinct roles in the antitumor response (Figure 2I). Flow cytometric analysis confirmed a significant reduction in the population of NK cells, CD4^+^ T cells, and CD8^+^ T cells in the spleen following intraperitoneal injection of anti‐NK1.1, anti‐CD4 or anti‐CD8 antibodies (Figure S1, Supporting Information). The depletion of CD8^+^ T cells in the combined treatment group significantly increased the growth of rechallenged tumors compared with the depletion of NK cells or CD4^+^ T cells, while the rechallenged tumors in NK or CD4^+^ T‐cell‐depleted mice were not significantly different from the isotype control groups (Figure 2J,K). In contrast, the rechallenged tumors in the combined treatment group with depleted NK or CD4^+^ T cells were smaller than those in untreated mice (Figure 2J,K). These data indicate that CD8^+^ T cells, as opposed to NK cells or CD4^+^ T cells, are the critical cellular components contributing to antitumor immunity and play a pivotal role in preventing tumor recurrence induced by the combination of MWA and Flt3L treatment. Ultimately, to ascertain the augmented tumor cytotoxicity of CD8^+^ T cells induced by the combined therapeutic approach, an *ex vivo* T‐cell‐mediated tumor cell lysis assay was conducted (Figure 2L). Strikingly, the CD8^+^ T cells isolated from the group receiving the combined treatment demonstrated the highest level of tumor‐killing efficacy (Figure 2M,N). In aggregate, the comprehensive dataset highlighted the instrumental role of CD8^+^ T lymphocytes in mitigating tumor recurrence in the LLC tumor rechallenge murine model.

### 2.3 Antitumor Immunity Depends on Central Memory T‐Like Cells Within TdLN

**Figure 3:**
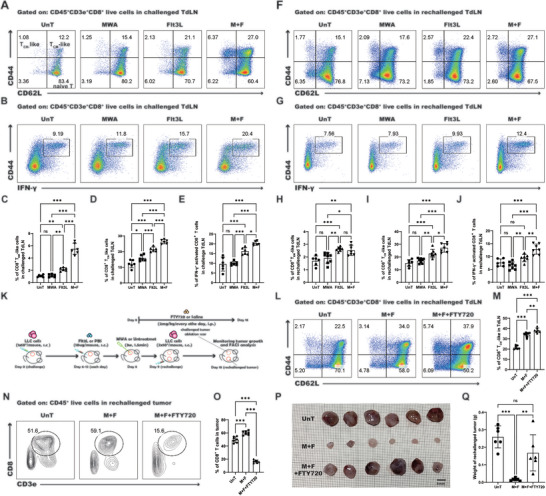
The combined treatment enhances the antitumor effect in a manner dependent on T_CM_‐like cells. A) Representative dot plot showing the percentages of T_EM_‐like (CD44^+^CD62L^−^) and T_CM_‐like (CD44^+^CD62L^+^) cells in the challenged TdLN. B) The proportion of IFN‐γ^+^ activated CD8^+^ T cells in the challenged TdLN, as shown in the flow cytometry dot plot. C–E) Statistical evaluations of the ratios of T_EM_‐like cells, T_CM_‐like cells, and IFN‐γ‐positive activated CD8^+^ T cells in the challenged TdLN across different treatment modalities. F) Representative flow cytometry plots showing the percentages of T_EM_‐like and T_CM_‐like cells in the rechallenged TdLN. G) The proportion of IFN‐γ^+^ activated CD8^+^ T cells in the rechallenged TdLN, as shown in flow cytometry plots. H–J) Statistical evaluations of the ratios of T_EM_‐like, T_CM_‐like cells, and IFN‐γ‐positive activated CD8^+^ T cells in the rechallenged TdLN across different treatment modalities. K) Schematic illustration of the experimental design for L–Q. L) The proportion of CD8^+^ T cells in the rechallenged TdLN after FTY720 blockade, as shown in flow cytometry plots. M) Statistical findings for L. N) The proportion of T_CM_‐like cells in the rechallenged TdLN after FTY720 blockade, as shown in flow cytometry plots. O) Statistical findings for N. P) Images of the rechallenged tumors after FTY720 blockade. Q) Statistical findings for P. Each group consisted of 6–8 mice, and the data were acquired from at least two independent experiments. Statistical significance was determined via one‐way ANOVA followed by Tukey's multiple comparisons test via GraphPad Prism 9.0.0 software (^*^ *p* < 0.05, ^**^ *p* < 0.01, ^***^ *p* < 0.001).

Central memory T (T_CM_)‐like cells have demonstrated the capability to induce robust antitumor immunity and exhibit enhanced persistence when compared to effector memory T (T_EM_) cells and effector T (Teff) cells.^[^ ^51^, ^52^, ^53^, ^54^ ^]^ To explore whether the combined treatment of MWA and Flt3L therapy promotes T_CM_‐like antitumor response, we assessed the frequency of CD8^+^CD44^+^CD62L^+^ T cells in the TdLN. CD44 and CD62L serve as critical surface markers for distinguishing T_CM_ and T_EM_ cell populations.^[^ ^55^ ^]^ Our study elucidated that the proportion of T_CM_‐like and T_EM_‐like cells within the challenged TdLN (**Figure** **3**A,C,D) and the percentage of T_CM_‐like cells in the rechallenged TdLN (Figure 3F,I) were significantly increased after the administration of the combined therapeutic regimen. Moreover, the capacity of activated T cells to produce IFN‐γ was substantially enhanced (Figure 3B,E,G,J). These results imply that the combination of MWA and Flt3L treatment potently enhances the activation and functional capabilities of T_CM_‐like cells within the TdLN.

To ascertain the role of T_CM_‐like cells in the antitumor efficacy of combined therapy, we utilized FTY720^[^ ^56^, ^57^ ^]^ within the LLC tumor rechallenge murine model to inhibit lymphocytes egress from the lymph node (Figure 3K). Following FTY720 blockade, our findings revealed a marked increase in the frequency of T_CM_‐like cells within the TdLN (Figure 3L,M), accompanied by a significant reduction in the proportion of CD8^+^ T cells within the tumor (Figure 3N,O). This suggests that T_CM_‐like cells are being retained within the TdLN, potentially impeding their migration to the tumor site. Notably, the inhibition of FTY720 markedly abated the therapeutic impact of the combined treatment on tumor growth (Figure 3P,Q). These findings suggest that T_CM_‐like cells in the TdLN play a critical role in mediating the antitumor response induced by MWA in combination with Flt3L therapy in lung cancer.

### 2.4 Tumor‐Specific Memory T‐Cell Responses are Initiated Within TdLN

**Figure 4:**
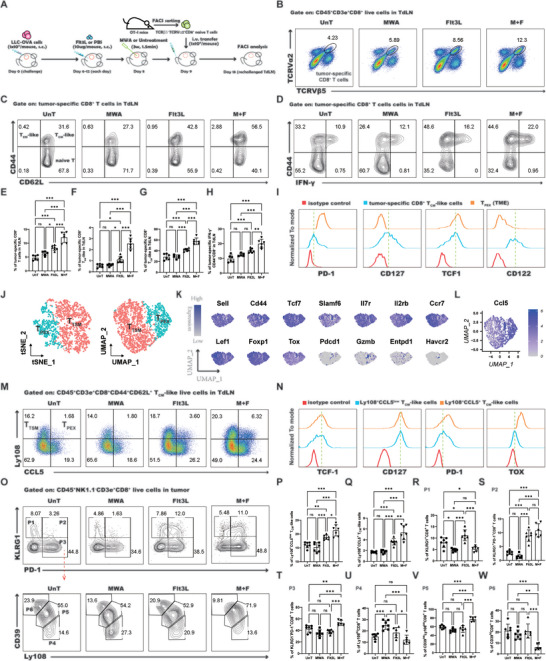
The combined treatment promoted the T_TSM_ cell response in the TdLN. A) Schematic illustration of the experimental design for B‐I. B) Fraction of tumor‐specific CD8^+^ T cells in the TdLN, as represented in the flow cytometry dot plot. C) Representative flow cytometry plots of the percentage of tumor‐specific CD8^+^ TCM‐like cells in the TdLN. D) The percentage of IFN‐γ^+^ tumor‐specific CD8^+^ T cells in the TdLN, as illustrated in the flow cytometry dot plot. E–H) Statistical findings for B, C, and D. I) The median fluorescence intensity (MFI) of PD‐1, CD127, TCF‐1, and CD122 expression on tumor‐specific CD8^+^ T_CM_‐like (blue), T_PEX_ (orange), and isotype control (red). J) Reclustering of cDCs and T_CM_‐like cells was performed via UMAP and tSNE analysis. K) Single‐cell transcription levels of representative genes illustrated in the TSNE plot for T_TSM_ and T_PEX_ cells from J. Gray, not expressed; blue, expressed. L) Single‐cell transcription level of *Ccl5* illustrated in the UMAP plot in J. M) Representative flow cytometry dot plot of the percentage of T_CM_‐like cells in the TdLN of the LLC tumor rechallenged murine model. N) The median fluorescence intensity (MFI) of TCF‐1, CD127, PD‐1, and TOX expression on endogenous Ly108^+^CCL5^low^ T_CM_‐like (blue), endogenous Ly108^+^CCL5^+^ T_CM_‐like (orange), and isotype control (red). P, Q) Statistical findings for M. O) Proportion of exhausted CD8^+^ T cells in the rechallenged TME, as displayed in a flow cytometry dot plot (P1: Teff, P2: KLRG1^+^PD1^+^ cells, P3: exhausted T cells, P4: T_PEX_, P5: T_EX_‐int, P6: T_EX_). The red arrow denotes the gating strategies for the subsequent step. R–W) Statistical results for M. Each group consisted of 6–8 mice, and the data were acquired from at least two independent experiments. Statistical significance was determined via one‐way ANOVA followed by Tukey's multiple comparisons test via GraphPad Prism 9.0.0 software (^*^ *p* < 0.05, ^**^ *p* < 0.01, ^***^ *p* < 0.001).

The persistence of an antitumor immune response is orchestrated by the enduring presence of CD8^+^ progenitor‐exhausted T (T_PEX_) cells, which function as a critical resource for the replenishment of effector T cells and ensure their abundance through self‐renewal.^[^ ^58^ ^]^ Research by Huang and colleagues has uncovered that tumor‐specific T_PEX_ cells arise from a lineage of T_TSM_cells. T_TSM_ cells represent a distinct population of tumor‐specific CD8^+^ T cells within TdLN that are characterized by the expression of canonical T_CM_‐associated markers, including IL‐7Ra (CD127), IL‐2Rb (CD122), and CD62L. Within TdLN, T_TSM_ cells are different from T_PEX_ cells by their reduced expression of PD‐1 and TOX. When compared to T_PEX_ cells, T_TSM_ cells demonstrate a superior ability to inhibit tumor growth upon adoptive transfer.^[^ ^59^ ^]^ Given the significant increase in T_CM_‐like cells within the TdLN following combined treatment, we utilized the OT‐I transfer model to investigate whether the combined therapy elicits antitumor immunity mediated by T_TSM_ cells. The OT‐I transfer model is a classical system used to observe antigen‐specific CD8^+^ T cell responses, wherein OT‐I^+^CD8^+^ T cells possess a transgenic TCR (Vα2/Vβ5) capable of specifically recognizing the ovalbumin (OVA)‐derived peptide.^[^ ^60^ ^]^ Building upon this model, we transferred naïve OT‐I^+^CD8^+^ T cells into a LLC‐OVA syngeneic murine model, in which LLC cells secrete OVA antigen (**Figure** **4**A). After combined MWA and Flt3L treatment, a substantial increase in the proportion of tumor‐specific CD8^+^ T cells (CD8^+^TCRVα2^+^TCRVβ5^+^) in the TdLN relative to those in the other groups was observed (Figure 4B,E). Additionally, among the tumor‐specific CD8^+^ T cells, a significant increase in the proportion of T_CM_‐like cells (Figure 4C,G) and the secretion of IFN‐γ (Figure 4D,H) was observed. As anticipated, our findings elucidated that the tumor‐specific T_CM_‐like cells exhibited the hallmark characteristics of T_TSM_, marked by high expression levels of CD127, TCF‐1, and CD122, complemented by strikingly low expression levels of PD‐1 (Figure 4I). The data indicate that the combined treatment significantly promotes the differentiation of T_TSM_ cells within TdLN.

### 2.5 Endogenous T_TSM_‐Cell Responses are Initiated Within TdLN

Flt3L‐based immunotherapy has the potential to enhance the oligoclonal expansion of host T cells and to promote the infiltration of tumors by antigen‐specific endogenous T cells (host‐derived and not exogenously adoptively transferred).^[^ ^60^ ^]^ Consequently, to ascertain whether combined treatment can amplify endogenous T_TSM_ cells, we performed single‐cell RNA sequencing (scRNA‐seq) on CD45^+^CD19^−^Ly6G^−^ cells sorted from TdLN in LLC tumor rechallenge murine model via the 10× Genomics platform and ultimately identified nine distinct cell populations (Figure S2A, Supporting Information). These populations were characterized based on their signature gene expression patterns (Figure S2B, Supporting Information): CD4^+^ naïve T cells (*Cd3e* ^+^ *Cd4* ^+^ *Sell* ^+^), CD4^−^CD8^−^ T cells (*Cd3e* ^+^ *Cd4* ^−^ *Cd8a* ^−^CD44^+^), Tregs (*Cd3e* ^+^ *Cd4* ^+^ *Foxp3* ^+^), CD8^+^ naïve T cells (*Cd3e* ^+^ *Cd8a* ^+^ *Sell* ^+^), CD8^+^ T_CM_‐like cells (*Cd3e* ^+^ *Cd8a* ^+^ *Cd44* ^+^ *Sell* ^+^ *Tcf7* ^+^), B cells (*Cd19* ^+^), NK cells (*Cd3e* ^−^ *Ncr1* ^+^), cDCs (*Itgax* ^+^ *Zbtb46* ^+^ *CD74* ^+^), and pDCs (*Bst2* ^+^ *Siglech* ^+^). Among them, the CD8^+^ T_CM_‐like cells in the TdLN expressed canonical T_CM_‐associated markers,^[^ ^61^ ^]^ including *Il7r* (CD127), *Il2rb* (CD122) and *Sell* (CD62L), *Ccr7*, *Tcf7*, *Lef1*, and *Foxp1* (Figure S2C, Supporting Information). Analysis of the cell population ratios revealed that the proportion of CD8^+^ T_CM_‐like cells in the combined treatment group was significantly greater than that in the other groups (Figure S2D, Supporting Information), which is consistent with our earlier findings. Therefore, the scRNA‐seq data also indicated that MWA combined with Flt3L treatment effectively promoted the differentiation of TdLN‐T_CM_‐like cells. Furthermore, through refined reclustering of the T_CM_‐like cells, we discerned two distinct subsets (Figure 4J). Analysis of the signature genes within these subsets revealed one population characterized by TCF‐1^+^ T_TSM_‐like cells with low expression of PD‐1 and TOX, while the other subset comprised TCF‐1^+^ T_PEX_ cells that were positive for PD‐1 and TOX (Figure 4K). These two subsets were differentiated by the expression levels of CCL5 (Figure 4L). These findings imply that the endogenous T_CM_‐like cell compartment harbors T_TSM_‐like cells. Next, we delved into the changes occurring within the endogenous T_TSM_‐like cells in the TdLN, revealing that these T_CM_‐like cells could be segmented into four distinct subpopulations based on the expression patterns of Ly108 (acting as a surrogate for TCF‐1) and CCL5 (Figure 4M). The Ly108^+^CCL5^low^ T_CM_‐like cells displayed a pronounced expression of CD127 and TCF‐1, while keeping the levels of PD‐1 and TOX comparatively low (Figure 4N), a phenotype that is in concordance with T_TSM_‐like cells. Notably, there was a significant increase in the percentage of these cells following the combined treatment (Figure 4P). Moreover, the Ly108^+^CCL5^+^ T_CM_‐like cells were characterized by a high expression of CD127, TCF‐1, PD‐1, and TOX (Figure 4N), a phenotype of T_PEX_ cells, with a concomitant significant rise in their proportion after the combined treatment. These findings collectively suggest that the combined treatment effectively promotes the differentiation of endogenous T_TSM_ cells.

### 2.6 The Combination Therapy Elicits a Marked Alteration of TME

To elucidate the impact of the combined treatment on exhausted T cells within TME, we employed flow cytometry to analyze the subsets of tumor‐infiltrating CD8^+^ T cells. In tumors, CD8^+^ T cells can differentiate into terminal effector (Teff, KLRG1^+^PD1^−^) cells and exhausted T cells (KLRG1^−^PD1^+^) according to their expression of KLRG1 and PD‐1. Exhausted T cells can be further categorized into T_PEX_ cells (TCF‐1^+^CD39^−^) and terminally exhausted CD8^+^ T (T_EX_) cells (TCF‐1^−^CD39^+^) based on the expression of TCF‐1 and CD39.^[^ ^62^ ^]^ Our findings demonstrated that Teff cells were markedly more prevalent in the untreated and Flt3L‐treated groups (Figure 4O,R), while KLRG1^+^PD‐1^+^ cells were significantly elevated in the Flt3L‐treated and the combined treatment groups compared to the untreated and MWA‐treated groups (Figure 4O,S). Notably, the combined treatment group exhibited the highest frequency of exhausted T cells (Figure 4O,T). Upon further stratification of the exhausted T cells based on the expression levels of Ly108 and CD39, we observed that T_PEX_ cells were more abundant in the MWA‐treated group relative to the other groups (Figure 4O,U), Ly108^int^CD39^int^ cells were significantly more prevalent in the combined treatment group compared to the other groups (Figure 4O,V), and T_EX_ cells were least frequent in the combined treatment group (Figure 4O,W). The data suggests that the combined treatment alters the composition of the tumor‐infiltrating CD8^+^ T cell subsets. Research has demonstrated that KLRG1^+^PD‐1^+^ T cells, noted for their active production of significant quantities of TNF‐α, IFN‐γ, perforin, and granzyme B, are indicative of a potent and non‐exhausted functional state.^[^ ^63^ ^]^ Furthermore, Ly108^int^CD39^int^ cells represent a transitional state between T_PEX_ and T_EX_, known as T_EX_‐int,^[^ ^64^, ^65^, ^66^, ^67^ ^]^ and have demonstrated enhanced functional capabilities relative to T_EX_, including the secretion of higher levels of IFN‐γ, TNF‐α, and IL‐2.^[^ ^65^, ^68^, ^69^ ^]^ Therefore, the increase in KLRG1^+^PD‐1^+^ and Ly108^int^CD39^int^ cells may underlie the suppression of tumor growth by the combined treatment. In aggregate, the combination of MWA with Flt3L treatment not only augments the differentiation of both T_TSM_ and T_PEX_ cells within the TdLN but also orchestrates remodeling of the exhaustion phenotype of CD8^+^ T cells within the TME, ultimately leading to the inhibition of tumor growth.

### 2.7 Migratory cDC1s Initiate the T_TSM_‐Mediated Antitumor Response

**Figure 5:**
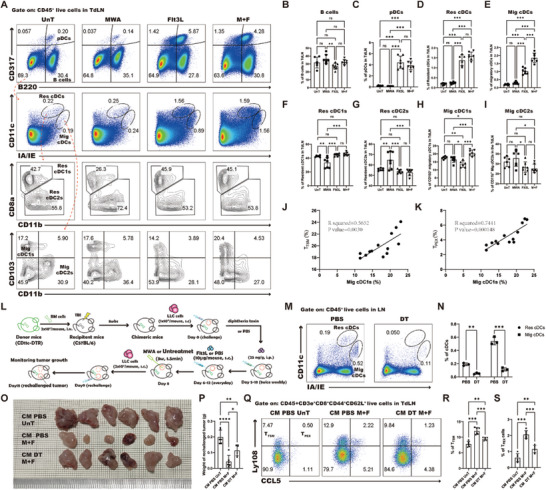
The Mig cDC1s are crucial for the T_TSM_‐mediated antitumor response. A) Representative flow cytometry dot plot of the percentages of B cells, pDCs, and cDC subtypes following various treatment regimens in the TdLN. The red arrow denotes the gating strategies for the subsequent step. B–I) Statistical results for the percentage of B cells, pDCs, Res cDCs, Mig cDCs, Res cDC1s, Res cDC2s, Mig cDC1s, and Mig cDC2s. J) Identification of the correlation between Mig cDC1s and T_TSM_ cells in the rechallenged TdLN. K) Identification of the correlation between Mig cDC1s and T_PEX_ cells in the rechallenged TdLN. L) Schematic illustration of the experimental design for M–S. M) Representative flow cytometry dot plot of the percentage of cDCs in the lymph nodes after DT treatment. N) Statistical results for M. O) Images of the rechallenged tumors in all groups. “CM PBS UnT” stands for CD11c‐DTR chimeric mice that received no treatment. “CM PBS M+F” refers to CD11c‐DTR chimeric mice that received combination treatment with MWA and Flt3L. “CM DT M+F” stands for CD11c‐DTR chimeric mice that received MWA, Flt3L, and DT treatment. P) Statistical results for O. Q) Representative flow cytometry dot plot of the percentage of T_TSM_ and T_PEX_ cells. R, S) Statistical results for Q. Each group consisted of 6–8 mice and the data were acquired from at least two independent experiments. Statistical significance was determined via one‐way analysis of variance (ANOVA) followed by Tukey's multiple comparisons test, and correlation analysis was conducted via Pearson's test via GraphPad Prism 9.0.0 software (^*^ *p* < 0.05, ^**^ *p* < 0.01, ^***^ *p* < 0.001).

Flt3L is a critical hematopoietic cytokine that plays a pivotal role in the development of antigen‐presenting cells (APCs) in murine models, including B cells and DCs.^[^ ^70^ ^]^ To clarify the role of APCs in antitumor immunity induced by the combination of MWA and Flt3L treatment, we analyzed the alteration of B cells, pDCs, and cDCs in the TdLN. The proportion of B cells remained unchanged following the combined treatment as compared to other groups (**Figure** **5**A,B). The frequency of pDCs significantly increased after Flt3L treatment, but that was not further augmented by the co‐administration of MWA (Figure 5A,C). These findings suggest that B cells and pDCs may not be the pivotal APCs responsible for the superior antitumor efficacy observed with the combined treatment compared to other treatments. Subsequently, we paid our attention to the alterations in cDCs following the combined treatment. In lymph nodes, cDCs constitute a heterogeneous cell population that can be delineated into two subsets: resident cDCs (Res cDCs) and migratory cDCs (Mig cDCs).^[^ ^71^ ^]^ Notably, flow cytometric analysis highlighted that Flt3L treatment, whether used alone or in conjunction with MWA, significantly enhanced the proportions of both Res and Mig cDCs compared to untreated and MWA‐treated controls (Figure 5A,D,E). However, no significant difference in the levels of Res cDCs was observed between Flt3L alone and combined treatment (Figure 5D). Furthermore, compared with Flt3L monotherapy, the combined treatment elicited a significant increase in the recruitment of Mig cDCs (Figure 5E). These findings implicate Mig cDCs as potentially pivotal cells in enhancing the antitumor immune response following combined treatment. Previous research has demonstrated that only Mig cDC1s are capable of uptaking TAAs and inducing antitumor immune responses after Flt3L monotherapy.^[^ ^60^ ^]^ Therefore, we investigated the changes in the cDC subsets after the combined treatment modality. Within the cDC subsets, Res cDCs can be further subdivided into resident cDC1s (Res cDC1s) and resident cDC2s (Res cDC2s), while Mig cDCs can be subdivided into migratory cDC1s (Mig cDC1s) and migratory cDC2s (Mig cDC2s).^[^ ^71^ ^]^ Following the combination intervention, the percentages of Res cDC1s, Res cDC2s, and Mig cDC2s showed no significant deviations when compared to both the untreated and Flt3L alone (Figure 5A,F,G,I). In contrast, the frequency of Mig cDC1s was significantly higher post‐treatment compared to other groups (Figure 5A,H). The aforementioned findings suggest that Mig cDC1s may indeed be the critical subset of cDCs that plays a key role in enhancing the antitumor immune response following combined treatment. To delineate the relationship between Mig cDC1s and the promotion of T_TSM_ or T_PEX_ cell differentiation, a positive correlation was noted between the proportion of Mig cDC1s and the ratio of T_TSM_ or T_PEX_ cells (Figure 5J,K). This additional evidence suggests that Mig cDC1s act as critical initiators of the T_TSM_‐mediated antitumor response after combined treatment.

Moreover, to validate the role of cDCs in the induction of antitumor immune responses following combined treatment, we developed a LLC tumor rechallenge murine model by employing CD11c‐DTR chimeric mice (Figure 5L). In the established model, administration of diphtheria toxin (DT) led to the efficient depletion of cDCs from the lymph nodes, encompassing both resident and migratory subsets (Figure 5M,N), while leaving the proportion of pDCs unaffected (Figure S3, Supporting Information). Once cDCs were eliminated, the combined treatment strategy compromised its inhibitory effect on the growth of the rechallenged tumor (Figure 5O,P), indicating a pivotal role for cDCs in orchestrating the antitumor immune response induced by the combined treatment. Furthermore, a remarkable reduction in the fraction of T_TSM_ or T_PEX_ cells in the TdLN was observed upon the elimination of cDCs (Figure 5Q–S), indicating that cDCs are crucial for the activation and differentiation of T_TSM_ cells.

### 2.8 ICOS‐Related Signaling was Enhanced Between Mig cDC1s and T_TSM_ Cells

**Figure 6:**
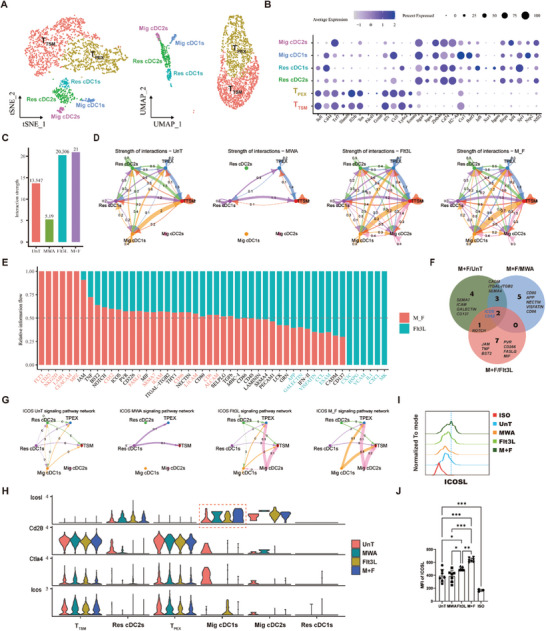
The combined treatment altered the cell–cell communication between Mig cDC1s and T_CM_‐like cells. A) Reclustering of cDCs and T_CM_‐like cells was performed via UMAP and tSNE analysis. B) Dot plot showing the relative average expression of a subset of marker genes across all clusters in A. C) The strength of cell–cell communication among cell populations. D) A circle plot was constructed to visualize the strength of cell–cell communication among cell populations. E) All significant signaling pathways were ranked based on their differences in overall information flow in the inferred networks between the Flt3L‐treated group and the combined treatment group. The top signaling pathways colored red are enriched in the combined treatment group, and these colored green are enriched in the Flt3L‐treated group. F) A Venn diagram delineates the top 10 enriched signaling pathways for intercellular interactions between the combined treatment group and the untreated group (M+F/UnT), the combined treatment group and the MWA treatment group (M+F/MWA), and the combined treatment group and the Flt3L treatment group (M+F/Flt3L), showing the intersections and unique aspects of the signaling pathways involved in cellular communication across these different experimental conditions. G) A circle plot was constructed to visualize the inferred ICOS signaling networks from the untreated group, MWA‐treated group, Flt3L‐treated group, and the combined treatment group. H) Expression distribution of the ICOS ligand and its receptor ICOS in different cell types. I) The median fluorescence intensity of ICOSL expression on Mig cDC1s in the TdLN following various treatment regimens. J) Statistical results for I. Each group consisted of 6–8 mice, and the data were acquired from at least two independent experiments. Statistical significance was determined via one‐way ANOVA followed by Tukey's multiple comparisons test via GraphPad Prism 9.0.0 software (^*^ *p* < 0.05, ^**^ *p* < 0.01, ^***^ *p* < 0.001).

To elucidate the mechanism by which Mig cDC1s maintain the niches of T_TSM_ cells in the TdLN following treatment with a combination of MWA and Flt3L. We performed reclustering of cDCs and T_CM_‐like cells from the scRNA‐seq dataset of TdLN, thereby identifying four subsets of cDCs: Res cDC1s, Res cDC2s, Mig cDC1s, and Mig cDC2s, as well as two subsets of T_CM_‐like cells: T_TSM_ and T_PEX_ cells (**Figure** **6**A,B). Through the application of CellChat, we examined the interactions among six cell subpopulations, including Mig cDC1s and T_CM_‐like cells. We observed a significant enhancement in the strength of cell‐cell interactions within the cohort receiving combined treatment (Figure 6C), particularly between Mig cDC1s and T_TSM_ or T_PEX_ cells (Figure 6D), when compared to the other treatment groups. To delve deeper into the discrepancies between the combined treatment group and other groups, the differential enrichment of overall cell–cell signaling was investigated (Figure 6E; Figure S4A,B, Supporting Information). We found that the top ten differentially upregulated signaling pathways shared two overlapping regions between the combined treatment group and other treatment groups (Figure 6F), namely CD52 and the inducible T‐cell costimulator (ICOS). Further analysis of the outputs of CD52 and ICOS signaling revealed that the CD52 signal is primarily conveyed from T_TSM_ and T_PEX_ to Mig cDC1s (Figure S4C, Supporting Information), whereas the ICOS signal is predominantly transmitted from Mig cDC1s to T_TSM_ and T_PEX_ (Figure 6G). These findings suggest that the induction of differentiation in T_TSM_ and T_PEX_ by the combined treatment may be mediated by the ICOS signaling from Mig cDC1s. By examining the changes in the transcription levels of ICOS‐related signaling genes, we discovered that *Icosl* on Mig cDC1s was significantly upregulated (Figure 6H). Consistent with these findings, flow cytometric analysis confirmed increased expression of the ICOS ligand (ICOSL) protein on CD103^+^ migrating cDC1s in the TdLN after a combination of MWA and Flt3L treatment (Figure 6I,J). Together, these results indicate that Mig cDC1s may promote the differentiation of T_TSM_ cells in the TdLN via the ICOS‐ICOSL signaling pathway.

### 2.9 Mig cDC1s Initiate T_TSM_ Cell Response via ICOS‐ICOSL Signaling Pathway

**Figure 7:**
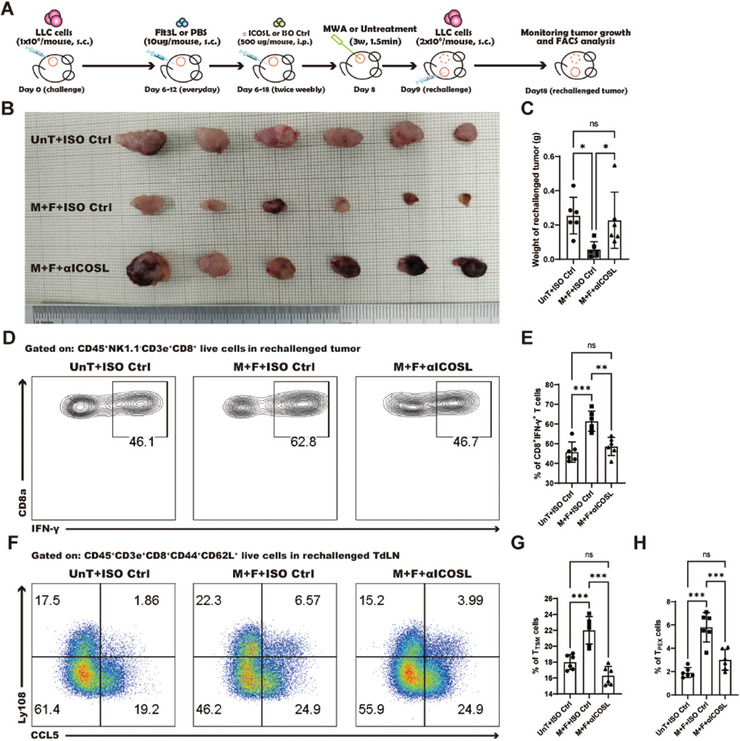
The combined treatment induced T_TSM_‐mediated antitumor response via the ICOS‐ICOSL axis. A) Schematic illustration of the experimental design for B‐H. B) Images of the rechallenged tumors in all treatment groups. C) Statistical results for B. D) The percentage of IFN‐γ^+^CD8^+^ T cells in the rechallenged tumor, as depicted in the flow cytometry dot plot. E) Statistical results for D. F) The percentage of T_TSM_ and T_PEX_ cells in the rechallenged TdLN, as visualized via a flow cytometry dot plot. G) Statistical results for the percentage of T_TSM_ cells. H) Statistical results for the percentage of T_PEX_ cells. Each group consisted of 6–8 mice, and the data were acquired from at least two independent experiments. Statistical significance was determined via one‐way ANOVA followed by Tukey's multiple comparisons test via GraphPad Prism 9.0.0 software (^*^ *p* < 0.05, ^**^ *p* < 0.01, ^***^ *p* < 0.001). “UnT+ISO Ctrl” refers to the IgG isotype control‐treated group. “M+F+ISO Ctrl” refers to MWA, Flt3L, and IgG isotype control‐treated groups. “M+F+αICOSL” refers to MWA, Flt3L, and anti‐ICOSL antibody‐treated groups.

To ascertain whether mig cDC1s facilitate the differentiation of TdLN‐T_TSM_ and TdLN‐T_PEX_ cells and mediate their antitumor immune response through the ICOS‐ICOSL signaling pathway, ICOSL monoclonal neutralizing antibodies were employed to block this pathway in LLC tumor rechallenge murine model (**Figure** **7**A). Our results demonstrated that blocking the ICOSL signaling pathway abrogated the inhibitory effect of the combined treatment on the growth of rechallenged tumors (Figure 7B,C). Moreover, the capacity of intratumoral CD8^+^ T cells to produce IFN‐γ was significantly reduced (Figure 7D,E). These findings suggest that interrupting the ICOSL signaling pathway leads to a loss of effectiveness of the combined therapy in reshaping the TME and enhancing T‐cell functionality. Additionally, following the blockade of the ICOSL signaling pathway, there was a significant reduction in the percentages of T_TSM_ and T_PEX_ cells within the TdLN (Figure 7F–H), indicating that the blockade of ICOSL signaling impairs the differentiation of T_TSM_ and T_PEX_ cells in the TdLN induced by the combined treatment. Collectively, these findings confirm that the integration of MWA with Flt3L activated the ICOS‐ICOSL costimulatory signaling pathway in Mig cDC1s, thereby enhancing the anti‐NSCLC immune response mediated by T_TSM_ and T_PEX_ cells.

### 2.10 Slc38a2 is Likely a Key Molecular for the Activation of Mig cDC1s

**Figure 8:**
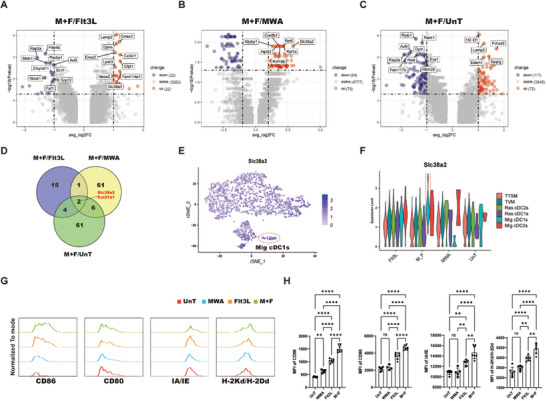
The combined treatment regimen induced alterations in the transcriptional profiles of Mig cDC1s. A–C) The volcano plot illustrates the differential genes in Mig cDC1s following combined treatment versus Flt3L treatment (M+F/Flt3L), combined treatment versus MWA treatment (M+F/MWA), and combined treatment versus untreatment (M+F/UnT). D) The Venn diagram illustrates the overlapping and distinctive upregulated gene interactions derived from A–C. E) Single‐cell transcription level of *Slc38a2* illustrated in the tSNE plot in T_CM_‐like cells and cDC subsets. F) Expression level of *Slc38a2* in different cell types following various treatments. G) The median fluorescence intensity of CD80, CD86, IA/IE, and H‐2Kd/H2‐Dd expression on Mig cDC1s in the TdLNs following various treatment regimens. H) Statistical results for G. Each group consisted of 6–8 mice, and the data were acquired from at least two independent experiments. Statistical significance was determined via one‐way ANOVA followed by Tukey's multiple comparisons test via GraphPad Prism 9.0.0 software (^*^ *p* < 0.05, ^**^ *p* < 0.01, ^***^ *p* < 0.001).

To elucidate the molecular mechanisms underlying the activation of Mig cDC1s by combined treatment, we conducted a transcriptional analysis of gene expression differences in Mig cDC1s following various treatments. We identified 22 genes upregulated more than two‐fold (log2FC > 1) with a significant difference (*p* < 0.05) in the combined treatment group compared to the Flt3L treatment group (**Figure** **8**A), 70 genes compared to the MWA treatment group (Figure 8B), and 73 genes compared to the untreated group (Figure 8C). By analysing the overlap of upregulated genes in the combined treatment group compared to other treatments, we found two intersection genes: *Slc38a2* and *Col27a1* (Figure 8D and Table S1–S3, Supporting Information). The expression of SLC38A2 in cDC1s is considered essential for maintaining the effector function of antigen‐specific CD8^+^ T cells within the TME.^[^ ^72^ ^]^ We observed that *Slc38a2* is widely expressed in Mig cDC1s and further analyzed changes of its expression following different treatments, finding that its transcriptional level was significantly higher after combined treatment than that in other groups (Figure 8E,F). Moreover, the expression level of SLC38A2 affects the expression of co‐stimulatory molecules in cDC1s.^[^ ^72^ ^]^ We further investigated changes in the expression of MHC‐II (IA/IE), CD86, CD80, and MHC‐I (H‐2Kd/H2‐Dd) on CD103^+^ Mig cDC1s following various treatments. Our results revealed that the expression of IA/IE, CD86, CD80, and H‐2Kd/H2‐Dd on CD103^+^ Mig cDC1s was significantly upregulated following treatment with the combination of MWA and Flt3L (Figure 8G,H). In conclusion, we propose that *Slc38a2* is likely a pivotal gene that mediates the activation of Mig cDC1s after combined treatment, drives the differentiation of T_TSM_ and T_PEX_ cells, and thereby enhances anti‐tumor immune responses.

### 2.11 Concurrent Therapeutic Strategies have Markedly Improved the Therapeutic Outcomes of PD‐1 Blockade‐Based Treatments for Lung Cancer

**Figure 9:**
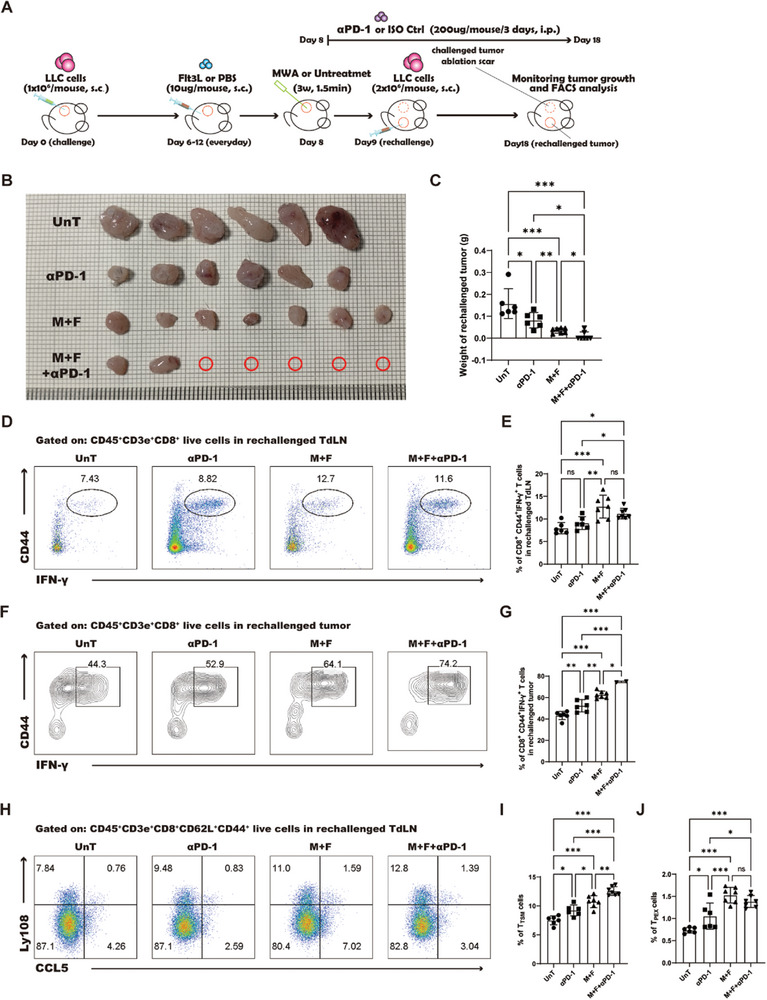
The combined treatment potentiates antitumor immunity in response to PD‐1 blockade. A) Schematic illustration of the experimental design for B‐J. B) Images of the rechallenged tumors in all treatment groups. C) Statistical results for B. D) The percentage of IFN‐γ^+^CD44^+^CD8^+^ T cells in the rechallenged TdLN, as depicted in the flow cytometry dot plot. E) Statistical results for D. F) The percentage of IFN‐γ^+^CD44^+^CD8^+^ T cells in the rechallenged TME, as depicted in the flow cytometry dot plot. G) Statistical results for F. H) The percentages of T_TSM_ and T_PEX_ cells in the rechallenged TdLN, as visualized via a flow cytometry dot plot. I) Statistical results for the percentage of T_TSM_ cells. J) Statistical results for the percentage of T_PEX_ cells. Each group consisted of 6–8 mice, and the data were acquired from at least two independent experiments. Statistical significance was determined via one‐way ANOVA followed by Tukey's multiple comparisons test via GraphPad Prism 9.0.0 software (^*^ *p* < 0.05, ^**^ *p* < 0.01, ^***^ *p* < 0.001).

The combination treatment of MWA and Flt3L has been observed to induce the differentiation of TdLN‐T_TSM_ cells, which were verified as bona fide responders to PD‐1/PD‐L1 blockade therapy.^[^ ^59^ ^]^ Therefore, this synergistic treatment regime holds the potential to augment the antitumor effects of PD‐1 blockade in lung cancer. To ascertain this, we concurrently administered PD‐1 blockade with the MWA and Flt3L triple therapy and evaluated the impact of this combined treatment on tumor growth (**Figure** **9A**). The results from the LLC tumor rechallenge murine model revealed that the combination of MWA and Flt3L significantly enhanced the tumor suppression achieved by PD‐1 blockade, with some rechallenged mice exhibiting no tumor growth, manifesting an exceptional antitumor efficacy (Figure 9B,C). Examination of IFN‐γ secretion by CD8^+^ T cells in the TdLN and TME indeed showed a significant increase in the proportion of IFN‐γ^+^ cells after MWA and Flt3L treatment in conjunction with PD‐1 blockade (Figure 9D–G), indicating a restoration of exhausted T cell function. Furthermore, the ratio of TdLN‐T_TSM_ and TdLN‐T_PEX_ cells was significantly higher in the group receiving triple therapy than that in those receiving either the combined treatment alone or PD‐1 blockade alone (Figure 9H–J), suggesting that PD‐1 blockade can further enhance the antitumor effects mediated by T_TSM_ induced by the MWA and Flt3L combination therapy. In conclusion, the MWA and Flt3L treatment regimen has been shown to enhance the responsiveness of lung cancer to PD‐1 blockade, thereby amplifying its therapeutic efficacy.

## 3 Discussion

Here, we combined local MWA therapy with Flt3L immunotherapy to treat NSCLC in a murine model. This approach differs from recent studies that have focused on combining MWA with ICIs, which generally act by obstructing T‐cell exhaustion within the TME to exert antitumor activity.^[^ ^25^ ^]^ However, as the number of infiltrating T cells in the tumor reaches a sufficient level, their responsiveness often diminishes.^[^ ^73^ ^]^ The deficiency of DCs in the TME is a pivotal determinant of reduced T‐cell infiltration. Our strategy augmented the population of DCs, specifically Mig cDC1s, which facilitated the activation of a greater number of naïve T cells into effector cells. This resulted in an augmented pool of activated T cells within the TdLN, thereby continuously replenishing the TME with antitumor‐active CD8^+^ T cells and converting “cold” tumors into “hot” ones. Notably, our findings demonstrated that the immune enhancement elicited by MWA in conjunction with Flt3L treatment significantly suppressed the growth of rechallenged tumors, indicating the potential to mitigate local recurrence and distant metastasis, which could offer substantial benefits for lung cancer patients.

In murine model, it is necessary to establish the syngeneic murine model that involve the injection of immunologically compatible cancer cells into immunocompetent mice.^[^ ^74^ ^]^ The only reproducible syngeneic model for lung cancer to date is the LLC model.^[^ ^75^ ^]^ Based on the LLC model, our objective was to investigate the therapeutic potential of combined treatment for preventing tumor recurrence after ablation by establishing the LLC tumor rechallenge murine model refer to previous research.^[^ ^76^, ^77^ ^]^

Flt3L is a critical cytokine for the differentiation and maturation of B cells, pDCs, and cDCs.^[^ ^70^ ^]^ It can modulate the antitumor efficacy of NK cells, as well as CD4^+^ and CD8^+^ T cells.^[^ ^78^, ^79^ ^]^ Our study elucidated that the activation of antitumor immune responses triggered by the combination of MWA with Flt3L treatment was contingent upon CD8^+^ T cells and Mig cDC1s, rather than B cells, pDCs, NK cells, or CD4^+^ T cells. The observed phenomenon is attributable to the elevation of TAAs after MWA therapy, which in turn promotes the efficient uptake of antigens by Flt3L‐expanded Mig cDC1s. This sequence of events leads to the maturation, activation, and augmented antigen‐presenting capacity of Mig cDC1s, thereby facilitating the cross‐presentation of TAAs to nave CD8^+^ T cells and triggering the initiation of CD8^+^ T‐cell‐mediated antitumor immune responses. The increased secretion of IFN‐γ by CD8^+^ T cells within the tumors after combined treatment, as well as the stronger tumor‐killing efficacy demonstrated by the sorted CD8^+^ T cells from tumors post‐combined treatment, validates this hypothesis.

The concept that the TdLN serves as a reservoir for systemic immune surveillance is increasingly being recognized.^[^ ^65^, ^80^, ^81^, ^82^ ^]^ Our findings have demonstrated that the combination of MWA with Flt3L treatment not only enhanced the function of CD8^+^ T cells but also increased the population of CD44^+^CD62L^+^ T_CM_‐like cells within the TdLN. Single‐cell sequencing results further confirmed that T_CM_‐like cells expressed canonical T_CM_‐associated markers, including *Il7r*, *Il2rb*, *Sell*, *Ccr7*, *Tcf7*, *Lef1*, and *Foxp1*. The blockade of T_CM_‐like cell migration in the TdLN by FTY720 led to the failure of the combined treatment to suppress tumor growth, indicating that the antitumor efficacy induced by the combined treatment is dependent on the TdLN and the T_CM_‐like cells within it. These observations underscore the critical role of TdLN in antitumor immune responses, emphasizing the therapeutic potential of targeting TdLN to prevent tumor recurrence and metastasis. In the OT‐I transfer model, which is widely recognized as a traditional framework for investigating the functions of tumor‐specific CD8^+^ T cells,^[^ ^83^ ^]^ we found that tumor‐specific T_CM_‐like cells in the TdLN expressed CD44, CD62L, CD127, and TCF‐1, while maintaining low expression of PD‐1, a phenotype consistent with the recently identified TdLN‐T_TSM_ cell.^[^ ^59^ ^]^ The proportion of tumor‐specific T_CM_‐like cells in the TdLN was significantly increased after combined treatment, suggesting that the combined treatment promotes the differentiation of T_TSM_ cells. TdLN‐T_TSM_ cells can mediate a strong antitumor immune response, and when transferred, they demonstrated greater antitumor therapeutic efficacy than T_PEX_ cells.^[^ ^59^, ^84^ ^]^ Therefore, the promotion of T_TSM_ cell differentiation by the combined treatment is an important factor in its ability to inhibit tumor recurrence. In the LLC tumor rechallenge murine model, we observed that T_CM_‐like cells could be further subdivided into four populations based on Ly108 and CCL5 expression. Ly108^+^CCL5^low^ T_CM_‐like cells exhibited a T_TSM_ cell phenotype, as observed by both longitudinal flow cytometric and scRNA‐seq analysis, while Ly108^+^CCL5^+^ T_CM_‐like cells displayed a T_PEX_ cell phenotype. The proportions of both T_TSM_ and T_PEX_ cells were increased significantly after combined treatment, which greatly aids in remodeling immunosuppressive TME. Following the combined treatment, there was a discernible shift in the differentiation trajectory of exhausted T cells within the TME, with T_PEX_, T_EX_, and T_EX_‐int delineating three distinct stages of exhausted T cell differentiation, among which T_EX_‐int emerged as the predominant subset. T_EX_‐int is a transitional state between T_PEX_ and T_EX_,^[^ ^64^, ^65^, ^66^, ^67^ ^]^ and it has been shown to possess superior functional capabilities compared to T_EX_, including the secretion of higher levels of IFN‐γ, TNF‐α, and IL‐2.^[^ ^65^, ^68^, ^69^ ^]^ This discovery underscores the notion that the combined treatment can transform the tumor microenvironment, shifting “cold” tumors to “hot” tumors, thereby heralding a promising strategy in immunotherapy.

DCs, particularly cDC1s, and their interaction with CD8^+^ T cells are pivotal in initiating antitumor immune responses.^[^ ^34^ ^]^ Among the factors involved, the upregulation of co‐stimulatory molecules such as CD40, CD80, CD86, and/or ICOSL on DCs is a critical step in stimulating T‐cell activation.^[^ ^30^ ^]^ Through cDCs depletion experiments, we have demonstrated that the differentiation of T_TSM_ and T_PEX_ cells induced by the combined treatment is dependent on cDCs. Furthermore, using single‐cell RNA sequencing, we discovered that the interaction signals between Mig cDC1s and T_TSM_ or T_PEX_ cells, particularly ICOS‐related signals, were increased following MWA combined with Flt3L treatment. The upregulation of ICOS‐related signals is attributed to the combined treatment‐induced enhancement of ICOSL expression on Mig cDC1s. ICOSL expression on activated APCs plays a critical costimulatory role in T‐cell activation.^[^ ^85^ ^]^ More importantly, ICOSL has been recognized as having potential functional importance in DC‐mediated (cross) priming of tumor‐specific T‐cell responses in vivo.^[^ ^86^ ^]^ Our findings indicate that inhibiting the ICOSL signaling pathway results in the inability of Mig cDC1s to effectively induce the differentiation of T_TSM_ and T_PEX_ cells after combined treatment, concurrently leading to the loss of tumor inhibition. These results imply that the combined treatment has the potential to amplify the interaction between Mig cDC1s and T_TSM_ or T_PEX_ cells by elevating ICOS signaling, consequently fostering the differentiation of T_TSM_ and T_PEX_ cells and boosting antitumor immune responses.

TdLN‐T_TSM_ cells are deemed to be the responders to PD‐1/PD‐L1 blockade therapy.^[^ ^59^ ^]^ Since the combined treatment can promote the differentiation of T_TSM_ cells, it theoretically stands to enhance the efficacy of tumor immunotherapy based on PD‐1 blockade. Our results demonstrate that when PD‐1 blockade is added to the combined treatment, rechallenged tumors exhibit almost no tumor growth, with the antitumor effects far surpassing those of either the combined treatment alone or PD‐1 blockade alone. Furthermore, we observed that after the triple therapy, the increase in the proportion of T_TSM_ cells within the TdLN was most significant, as well as an increase in the secretion of IFN‐γ by CD8^+^ T cells within the tumor. These findings suggest that the combination of MWA with Flt3L treatment indeed significantly boosts the efficacy of tumor immunotherapy based on PD‐1 blockade.

This study has several limitations. In this study, our data reveal that after the administration of combined treatment, there is a concurrent upregulation of SLC38A2 in Mig cDC1s, working in concert with CD80, CD86, MHC‐I, MHC‐II, and ICOSL. Although SLC38A2 has been reported to play a critical role in maintaining antigen‐specific CD8^+^ T cells induced by cDC1s,^[^ ^72^ ^]^ further confirmation is required in mice with conditional deletion of SLC38A2 in DCs to ascertain whether the antitumor immune response induced by combined treatment is dependent on SLC38A2 within DCs. Thus, due to the constraints of experimental conditions, we can only speculate that the upregulation of SLC38A2 in Mig cDC1s may contribute to the antitumor immune response induced by the combined treatment. Additionally, we employed ICOSL‐blocking antibodies to inhibit the ICOSL signaling pathway due to current technical limitations, rather than targeting the depletion of cDC1s. This method does not entirely exclude the possibility that other APCs might activate CD8^+^ T cells. Lastly, the study investigating the inhibition of recurrence post‐MWA when combined with Flt3L treatment is merely a preclinical investigation. The effectiveness of this strategy in terms of antitumor activity within clinical contexts or its ability to bolster the effectiveness of standard ICIs for NSCLC treatment is still unknown. However, MWA is already extensively applied in the clinical management of NSCLC, and phase I and II clinical trials for the use of Flt3L in conjunction with tumor adjuvant therapy have received approval.^[^ ^42^, ^43^ ^]^ Therefore, we are in a position to seize the opportunity and are planning to seek authorization for clinical trials to ascertain the safety and effectiveness of the combined MWA and Flt3L treatment regimen for advanced NSCLC in the coming years.

## 4 Conclusions

In conclusion, this study has demonstrated that the combined treatment of MWA and Flt3L can remodel the TME in a murine model by promoting the differentiation of T_TSM_ and T_PEX_ cells within the TdLN. This remodeling directs tumor‐infiltrating exhausted T cells to differentiate more toward a T_EX_‐int phenotype with enhanced antitumor activity, thereby significantly suppressing the recurrence of lung cancer after MWA. The antitumor immune response induced by the combined treatment is realized through the enhanced interaction between Mig cDC1s and T_TSM_/T_PEX_ cells, which is facilitated by the upregulation of ICOSL on Mig cDC1s. The mechanism underlying the upregulation of ICOSL may involve the increased transcription of SLC38A2 in Mig cDC1s. In summary, the combination of MWA and Flt3L potently activates T_TSM_ and T_PEX_ cell‐mediated antitumor immunity, indicating a novel strategy to enhance the response of solid tumors to immunotherapeutic interventions.

## 5 Experimental Section

### Animal

C57BL/6, CD11c‐DTR, and OT‐I TCR transgenic mice (6–8 weeks old) were purchased from Shanghai Model Organisms Center Inc. (Shanghai, China) and housed in a specific‐pathogen‐free (SPF) facility at Shandong First Medical University & Shandong Academy of Medical Sciences (Shandong, China). All animal experiments were conducted according to protocols approved by the Animal ethics committee of the Shandong First Medical University& Shandong Academy of Medical Sciences (NSFC: No. 2020–497).

### Cell Culture

Lewis lung carcinoma (LLC) and LLC/Luc cell lines were purchased from FuHeng Biology (Shanghai, China). The LLC‐OVA cell line was obtained from Shanghai Genechem Co., Ltd., China. All the cell lines were grown in complete DMEM‐10 medium: DMEM (Gibco, NY, USA), 10% (v/v) fetal bovine serum (FBS, Gibco, NY, USA), 100 U mL^−1^ penicillin, and 100 mg mL^−1^ streptomycin.

### LLC Tumor Rechallenges Murine Model

LLC cells (1 × 10^6^/mouse) were subcutaneously injected into the right axillary fossa of the mice on day 0. After the tumor reached a maximum diameter of ≈8–10 mm, the mice received different therapeutic interventions (≈8 days): untreated, MWA alone, Flt3L alone, or MWA plus Flt3L. MWA was conducted exclusively on the tumor located in the right axillary fossa on day 8. The procedure involved the percutaneous insertion of an ablation electrode into the deepest part along the longitudinal axis of the tumor. Treatments were administered for 1.5 min and 3 W. Two days before MWA treatment (≈day 6), Flt3L (Clone 1G9, BioXcell, NH, USA) was continuously administered at a dose of 10 µg per mouse via subcutaneous injection for 7 days (from day 6 to day 12). For tumor rechallenge, LLC/Luc cells (2 × 10^6^/mouse) were subcutaneously injected into the left axillary fossa of the mice on day 9. The growth of tumor in the left axillary fossa (rechallenged) was recorded every 2 days.

### Immune Cell Depletion

Beginning 2 days before MWA treatment, the mice were treated with an initial dose of 200 µg/mouse of anti‐CD4 (clone GK1.5, BioXCell, NH, USA), anti‐CD8 (clone 2.43, BioXCell, NH, USA) and anti‐NK1.1 (Clone PK136, BioXcell, NH, USA) antibodies in PBS, followed by similar dosing at 100 µg/mouse every 4 days throughout tumor growth cycle, and the mice belonging to the control group were administered control IgG.

### In Vivo Antibody Treatment

For monoclonal antibody blocking, a neutralizing mouse anti‐ICOSL antibody was purchased from BioXCell. Two days before MWA treatment, the mice were administered 500 µg of anti‐ICOSL antibodies (Clone HK5.3, BioXcell, NH, USA) by intraperitoneal injection twice a week, and this regimen was continued throughout the experiment. The control mice were simultaneously treated with IgG.

### Preparation of Single‐Cell Suspensions from Mouse Samples

Tumors were first cut into tiny pieces with scissors and then enzymatically digested with 1 mg mL^−1^ collagenase IV (Gibco, NY, USA) for 40 min at 37 °C. The tissues were filtered through a 70 µm cell strainer and resuspended in 40% Percoll (Cat. # 17‐0891‐01, MA, USA) for centrifugation at 1200 × g for 20 min at room temperature, followed by incubation with ACK buffer to lyse the erythrocytes. All the isolated cells were suspended in PBS supplemented with 2 mM EDTA and 1% FBS. The spleen was digested with 1 mg mL^−1^ of collagenase IV for 30 min, and the tumor‐draining lymph nodes (TdLNs) were digested for 15 min at 37 °C, both without Percoll treatment.

### Flow Cytometry

Surface markers were stained in PBS containing 2% BSA or FBS (w/v) at 4 °C for 30 min after Fc blocking (Invitrogen, Cat. # 14‐0161‐86, CA, USA) to characterize murine immune cell subsets. For the detection of cytokine production and CCL5, lymphocytes were stimulated for 5 h in a cell stimulation cocktail (eBioscience, Cat. # 00–4975, NY, USA). Intracellular cytokine staining (ICS) for IFN‐γ was performed via a Cytofix/Cytoperm Fixation/Permeabilization Kit (BD Biosciences, Cat. #554714, CA, USA). FVS700 (BD Biosciences, Cat. # 564997, CA, USA) was used to discriminate between viable and nonviable cells according to the manufacturer's instructions. The following reagents were purchased from BD Biosciences (CA, USA): CD45‐BV786 (Cat. # 564225), CD3e‐BUV737 (Cat. # 612771), PD‐1‐AF488 (Cat. # 568576), CD4‐APC/Cy7 (Cat. # 552051), CD8a‐BV650 (Cat. # 563234), CD44‐BV510 (Cat. # 563114), CD62L‐PE/CF594 (Cat. # 562404), Ly108‐AF647 (Cat. # 561547), CD127‐BV711 (Cat. # 565490), CD103‐biotin (Cat. # 557493), CD8a‐FITC (Cat. # 553030), CD80‐BUV737 (Cat. # 612773), CD86‐BUV395 (Cat. # 564199), CD19‐FITC (Cat. #557398) and Ly6G‐PerCP/Cy5.5 (Cat. # 560602). The following reagents were purchased from Invitrogen (CA, CA): NK1.1‐BUV395 (Cat. # 363‐5941‐82), KLRG1‐PerCP/eF710 (Cat. # 46‐5893‐82), TCR Vα2‐FITC (Cat. # 11‐5812‐82), TCR Vβ5‐eF450 (Cat. # 48‐5796‐82), CD11c‐PerCP/Cy5.5 (Cat. # 45‐0114‐82), B220‐SB600 (Cat. # 63‐0452‐82), CD11b‐APC/Cy7 (Cat. # A15390), Ly6C‐eF450 (Cat. # 48‐5932‐82) and H‐2Kd/H‐2Dd‐FITC (Cat. # 11‐5998‐82). The following reagents were purchased from BioLegend (CA, USA): CD39‐PE/Cy7 (Cat. # 143806), CCL5‐PE (Cat. # 149104), IFN‐γ‐PE (Cat. # 505808), IA/IE‐BV510 (Cat. # 107636), CD317‐BV711 (Cat. # 127039), streptavidin‐BV650 (Cat. # 405232) and ICOSL‐PE (Cat. # 107405).

### Cell Sorting and Adoptive T‐Cell Transfer

For the isolation of OT‐I ^+^CD8^+^ T cells, spleens and skin‐draining lymph nodes were harvested from OT‐I TCR transgenic mice following cervical dislocation, and single‐cell suspensions were prepared according to a previously described protocol. Single‐cell lymphocyte suspensions were first labeled with CD45‐BV786 and CD3e‐APC/Cy7 (BD Biosciences, Cat. #557596), CD8‐BV650, CD44‐BV510, CD62L‐PE/CF594, TCR Vα2‐FITC, TCR Vβ5‐eF450 and FVS700 (live/dead) before FACS analysis. CD45^+^CD3e^+^CD8^+^CD44^−^CD62L^+^TCR Vα2^+^TCR Vβ5^+^ live cells (naïve OT‐1^+^CD8^+^ T cells) were subsequently sorted via BD FACSAria Fusion (BD Biosciences) to achieve a purity greater than 95%, after which 1 × 10^6^ naïve OT‐1^+^CD8^+^ T cells were adoptively transferred into LLC‐OVA tumor‐bearing mice.

To sort CD8^+^ T cells from the TME, single‐cell lymphocyte suspensions from the tumors were first labeled with CD45‐BV786, CD3e‐APC/Cy7 (BD Biosciences, Cat. #557596), CD8‐BV650 and FVS700 (live/dead) before FACS analysis. CD45^+^CD3e^+^CD8^+^ live cells were subsequently sorted via BD FACSAria Fusion (BD Biosciences) to achieve a purity greater than 95%.

### CD11c‐DTR Chimeric Mice Model

Eight‐week‐old C57BL/6 mice received 11 Gy X‐ray irradiation before intravenous injection of CD11c‐DTR mouse BM cells (2 × 10^6^ total cells). Chimeric mice were used ≈2 months after reconstitution.

### Ex Vivo T‐Cell‐Mediated Tumor Cell Killing Assay

LLC cells were labeled with CellTrace Violet (Thermo Fisher, Cat. # C34557, MA, USA) according to the manufacturer's instructions. The complete medium was then used to resuspend LLC cells at a density of 1 × 10^5^ ml^−1^. CD8^+^ T cells were sorted from the TME and cocultured with labeled LLC cells in a 96‐well plate at a fixed effector–target ratio of 5:1 for 6 h. The cells were then washed twice with PBS and stained with FVS700 before being examined via flow cytometry.

### Immunohistochemical Analysis

Paraffin‐embedded tumor tissues were cut into 4 µm thick sections and mounted on poly L‐lysine‐coated slides. Staining was performed via an automatic immunohistochemistry machine (BenchMark ULTRA, Roche, Basel, Switzerland). The primary antibody used was anti‐mouse Ki67 (ABclonal, Wuhan, China), and the secondary antibody was horseradish peroxidase (HRP)‐conjugated goat anti‐rabbit IgG (ZSGB‐BIO, Beijing, China). DAB was used as the chromogen. Finally, hematoxylin counterstaining was performed. Tonsil tissue was used as a positive control, and PBS instead of the primary antibody was used as a negative control.

### 3′ Single‐Cell RNA‐Seq (scRNA‐Seq)

Single‐cell suspensions of the TdLNs were prepared via the method described above. The cells were enriched via a CD45 (TIL) Microbead Mouse Kit (Miltenyi Biotec, Cat. # 130‐110‐618, Bergisch Gladbach, Germany) according to the magnetically activated cell sorting (MACS) protocol and stained with CD19‐FITC, Ly6G‐PerCP/Cy5.5, CD45‐BV786 and FVS700 for FACS sorting. Approximately 1 × 10^6^ CD45^+^CD19^−^Ly6G^−^ live cells were sorted via a BD Aria Fusion instrument. For FACS analysis, single cells were sorted into flow tubes and cell viability was tested by determining the AOPI to ensure sufficient cell quality. Then, the cell suspension, which contained 300–600 living cells per microliter, as determined via CountStar (Shanghai, China), was loaded onto a chromium single‐cell controller (10× Genomics, CA, USA) to generate single‐cell gel beads in the emulsion according to the manufacturer's instructions. Single‐cell transcriptome amplification was performed via an S1000™ Touch Thermal Cycler (Bio‐Rad, CA, USA) at 53 °C for 45 min, followed by incubation at 85 °C for 5 min and holding at 4 °C. cDNA templates were generated and then amplified, and the quality was assessed via an Agilent 4200 instrument (performed by NovelBio Technology, Beijing, China).

### scRNA‐Seq Data Processing

The newly generated scRNA‐seq data obtained from 10× Genomics were aligned to the mm10 mouse reference genome and quantified via the Cell Ranger Single‐Cell Software Suite. The preliminarily filtered data generated by Cell Ranger were used for a Seurat object created via the R package Seurat (version 3.2.3). Doublets were removed via the Doublet Finder package. Further quality control was applied to cells via three metrics in a stepwise manner, including the total UMI count, number of detected genes, and proportion of the mitochondrial gene count per cell. Specifically, cells with more than 5000 UMI counts and 10% mitochondrial gene counts were excluded.

### Statistical Analysis

Statistical significance was determined via one‐way analysis of variance (ANOVA) followed by Tukey's multiple comparisons test, and correlation analysis was conducted via Pearson's test via GraphPad Prism 9.0.0 software (^*^ *p* < 0.05, ^**^ *p* < 0.01, ^***^ *p* < 0.001).

## Conflict of Interest

The authors declare no conflict of interest.

## Author Contributions

M.W., J.S., and F.X. contributed equally to this work and should be considered co‐first authors. M.W., J.S., and F.X. performed the experiments and analyzed the data with S.W., P.L., J.M., Z.C., and Q.X. M.W. processed and analyzed scRNA‐seq data. Q.X., Z.W., and X.Y. conceived, designed, and supervised the project and contributed equally to this work. M.W., J.S., F.X., and X.Y. wrote the manuscript, with all authors contributing to the revision of the manuscript.

## Supporting information



Supporting Information

Supplemental Table 1

Supplemental Table 2

Supplemental Table 3

## Data Availability

The data that support the findings of this study are available from the corresponding author upon reasonable request.
