# Supplementary material for: Microwave Ablation Combined with Flt3L Provokes Tumor‐Specific Memory CD8+ T Cells‐Mediated Antitumor Immunity in Response to PD‐1 Blockade
Source: Adv Sci (Weinh). 2024 Dec 4;12(4):2413181. doi: 10.1002/advs.202413181 (PMC11775548; doi:10.1002/advs.202413181)
Supplement: Supplementary file 1 — Supporting Information [file ADVS-12-2413181-s002.docx]

**Supplemental figures**


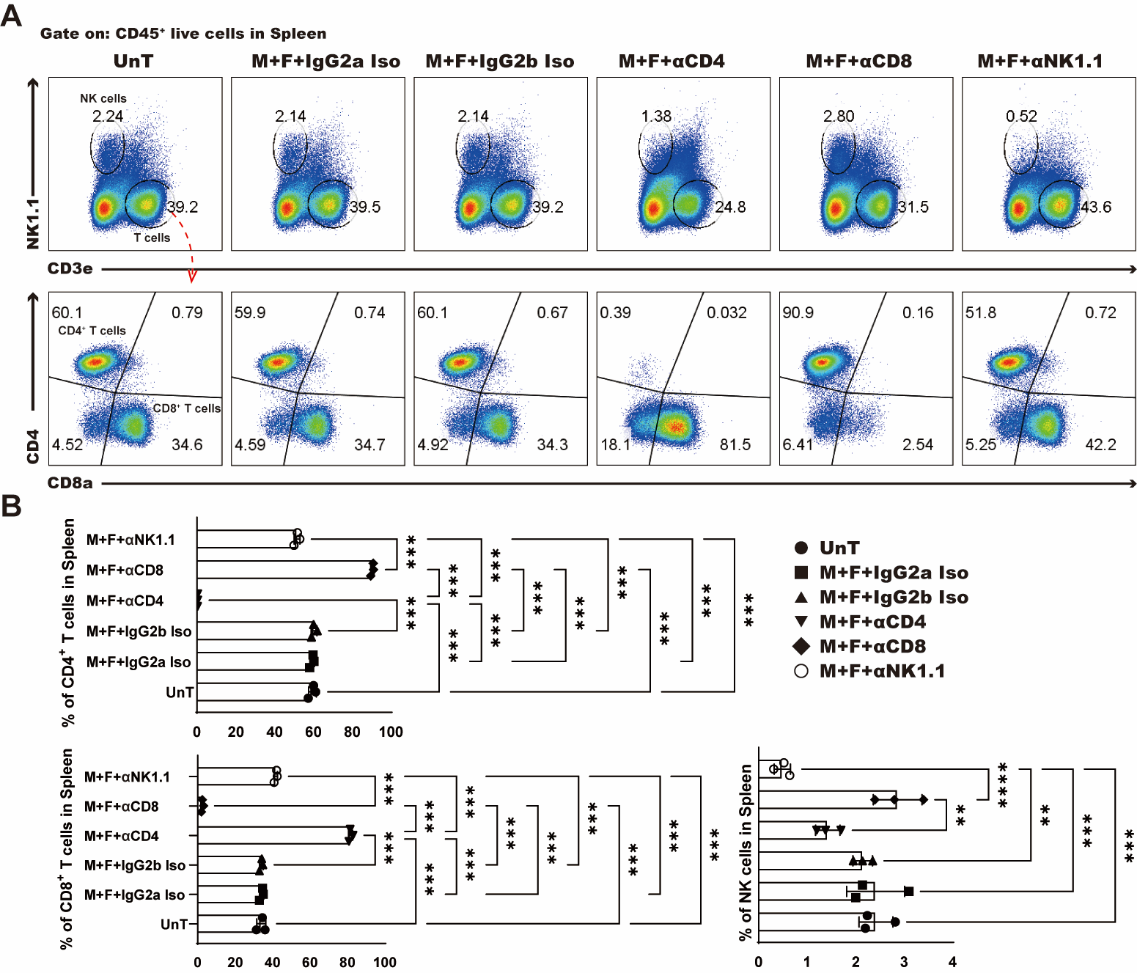


**Figure S1.** Depletion of NK cells, CD4^+^ T cells, and CD8^+^ T cells *in vivo*, related to Figure 2. A) Flow cytometric analysis was conducted to evaluate the alterations in the ratios of NK cells, CD4^+^ T cells, and CD8^+^ T cells within the spleen following the administration of anti-NK1.1, anti-CD4, anti-CD8α antibodies, as well as an IgG2a isotype control and an IgG2a isotype control monoclonal antibody. B) Statistical results for A.


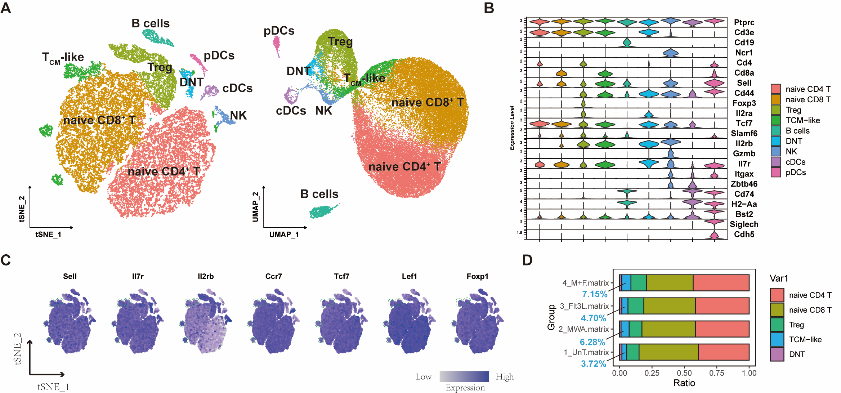


**Figure S2.** Transcriptional profiling of TdLN-derived CD45^+^CD19^-^Ly6G^-^ sorted cells by scRNA-seq, related to Figure 4 and Figure 6. A) UMAP and tSNE visualization of the scRNA-seq clusters of CD45^+^CD19^-^Ly6G^-^ sorted cells. B) StackedVlnPlot representing the relative average expression of a subset of marker genes across all clusters. C) Single-cell transcription levels of representative genes illustrated in the tSNE plot from A. D) Bar plot demonstrating percentages of T cell clusters as a fraction of total T cells for each group.


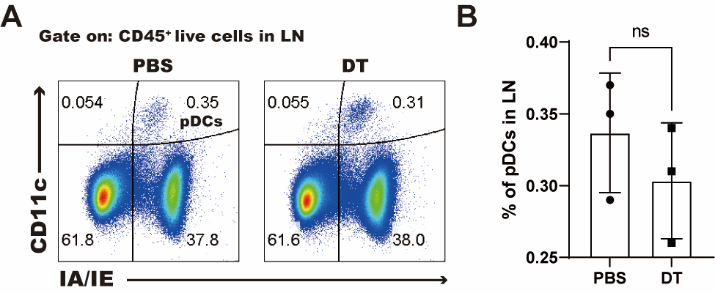


**Figure S3.** The percentage of pDCs in CD11c-DTR chimeric mice was found to be unaltered subsequent to DT treatment, related to Figure 5. A) Representative flow cytometry pseudocolor of the percentages of pDCs within LN. B) Statistical results for A.


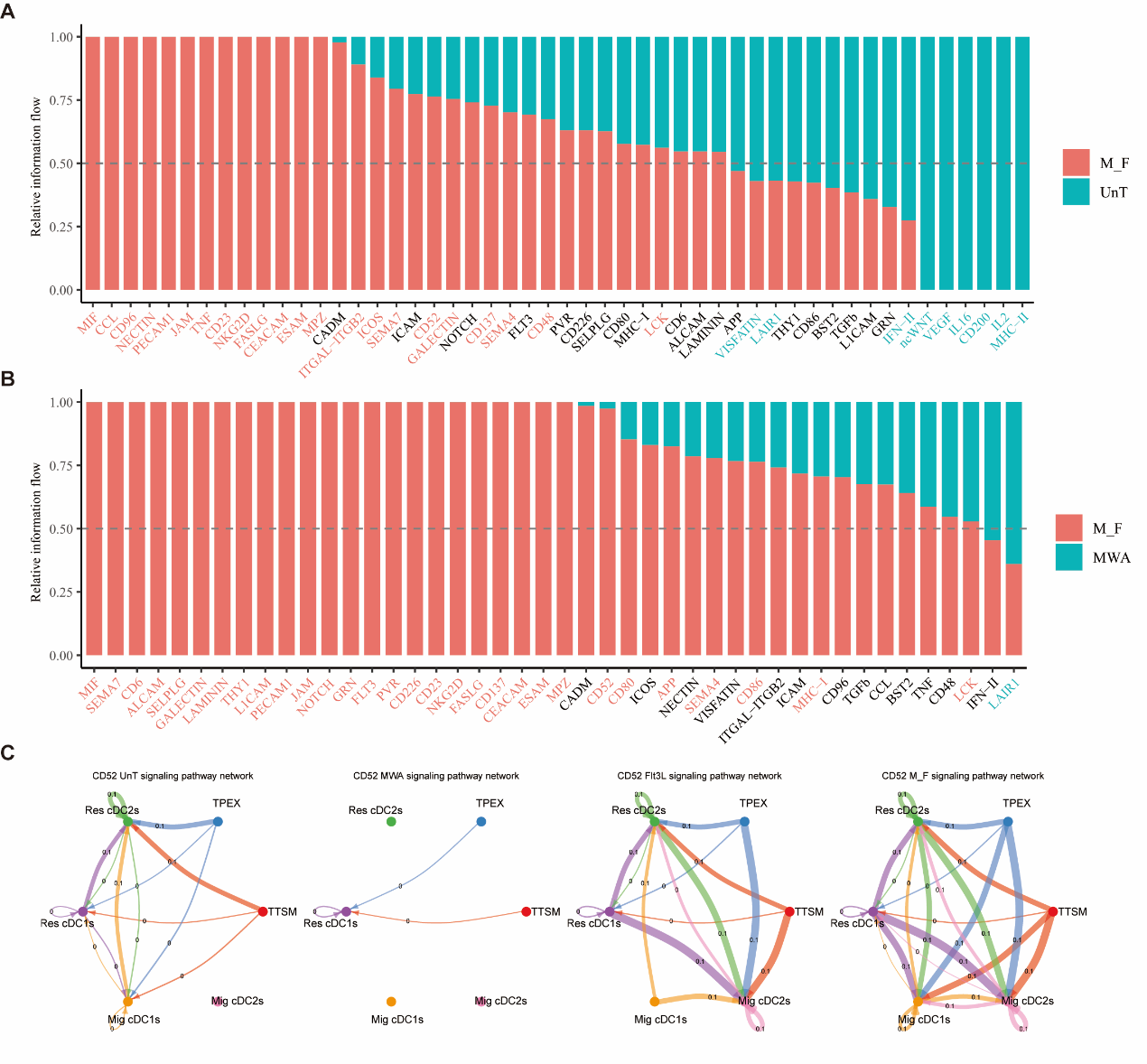


**Figure S4.** The cell-cell communication between cDC1 and T_CM_-like cell subsets, related to Figure 6. A) All significant signaling pathways were ranked on the basis of their differences in overall information flow in the inferred networks between the untreated group and the combined treatment group. B) All significant signaling pathways were ranked on the basis of their differences in overall information flow in the inferred networks between the MWA-treated group and the combined treatment group. C) A circle plot was constructed to visualize the inferred CD52 signaling networks from untreated group, MWA-treated group, Flt3L-treated group and the combined treatment group.


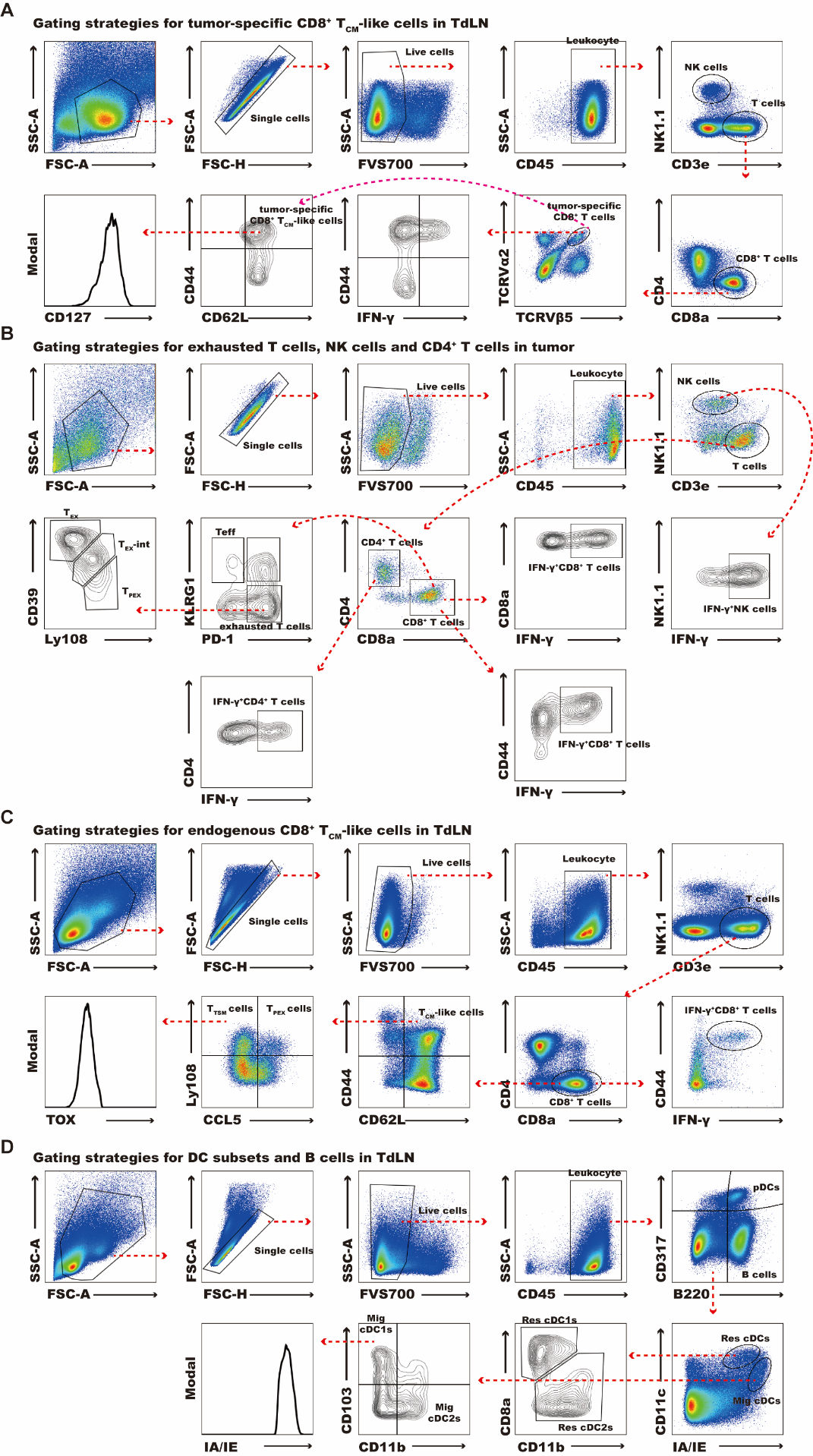


**Figure S5.** Gating strategies for the identification of tumor-specific and endogenous CD8^+^ T_CM_-like cells, DC subsets, B cells within TdLN, and NK cells, CD4^+^ T cells, exhausted T cells within the TME. A) Gating strategies for tumor-specific CD8^+^ T_CM_-like cells in TdLN, related to Figure 4. B) Gating strategies for NK cells, CD4^+^ T cells, exhausted T cells in TME, related to Figure 2 and 4. C) Gating strategies for endogenous CD8^+^ T_CM_-like cells within TdLN, and NK cells, related to Figure 3 and 4. D) Gating strategies for DC Subsets and B cells within TdLN, related to Figure 5.
